# Trastuzumab Alters the Expression of Genes Essential for Cardiac Function and Induces Ultrastructural Changes of Cardiomyocytes in Mice

**DOI:** 10.1371/journal.pone.0079543

**Published:** 2013-11-08

**Authors:** M. Khair ElZarrad, Partha Mukhopadhyay, Nishant Mohan, Enkui Hao, Milos Dokmanovic, Dianne S. Hirsch, Yi Shen, Pal Pacher, Wen Jin Wu

**Affiliations:** 1 Division of Monoclonal Antibodies, Office of Biotechnology Products, Office of Pharmaceutical Science, Center for Drug Evaluation and Research, U.S. Food and Drug Administration, Bethesda, Maryland, United States of America; 2 Interagency Oncology Task Force (IOTF) Fellowship: Program 4 - Cancer Prevention Fellow, National Cancer Institute, National Institutes of Health, Bethesda, Maryland, United States of America; 3 Laboratory of Physiologic Studies, National Institute on Alcohol Abuse and Alcoholism, National Institutes of Health, Bethesda, Maryland, United States of America; University of Western Ontario, Canada

## Abstract

Treatment with trastuzumab, a humanized monoclonal antibody directed against the extracellular domain of Human Epidermal Growth Factor Receptor **2** (HER2), very successfully improves outcomes for women with HER2-positive breast cancer. However, trastuzumab treatment was recently linked to potentially irreversible serious cardiotoxicity, the mechanisms of which are largely elusive. This study reports that trastuzumab significantly alters the expression of myocardial genes essential for DNA repair, cardiac and mitochondrial functions, which is associated with impaired left ventricular performance in mice coupled with significant ultrastructural alterations in cardiomyocytes revealed by electron microscopy. Furthermore, trastuzumab treatment also promotes oxidative stress and apoptosis in myocardium of mice, and elevates serum levels of cardiac troponin-I (cTnI) and cardiac myosin light chain-1 (cMLC1). The elevated serum levels of cMLC1 in mice treated with trastuzumab highlights the potential that cMLC1 could be a useful biomarker for trastuzumab-induced cardiotoxicity.

## Introduction

Treatment with trastuzumab, a humanized monoclonal antibody directed against the extracellular domain of Human Epidermal Growth Factor Receptor 2 (HER2), significantly improves outcomes for women with HER2-positive breast cancer [1-4]. Trastuzumab is clinically efficacious either as a single agent or in combination with standard chemotherapy regimens such as anthracyclines [1]. However, both anthracyclines and trastuzumab are associated with considerable cardiotoxicity [5-7]. Anthracycline-associated cardiotoxicity includes changes in myocardial ultrastructure, such as vacuolization and cardiomyocyte loss [5], which may lead to irreversible cardiomyopathy with a poor prognosis [8]. In contrast, trastuzumab-induced cardiotoxicity initially was thought to be reversible upon stopping treatment, and was considered not to be associated with ultrastructural changes [9,10]. Trastuzumab-induced cardiotoxicity manifests clinically as a decrease in left ventricular ejection fraction (LVEF) and heart failure [2-4,11], and was reported to occur in up to 7% of patients when trastuzumab is used as a single agent^6,7^. When combined with an anthracycline, cardiotoxicity is notably increased and has been reported to occur in up to 27% of patients [6]. 

Telli et al. reviewed several major adjuvant trastuzumab trials and reported that for every 30 women treated with trastuzumab, one would develop a cardiac event defined as the cardiac death or severe New York Heart Association (NYHA) class III/IV congestive heart failure (CHF) at three years, and one-in-five women treated with trastuzumab will have some form of cardiac dysfunction requiring discontinuation of treatment [11]. Based on the data obtained from these large clinical trials, the concept of the reversibility of trastuzumab-related cardiotoxicity is called into question [11]. In a larger population-based study involving multiple cancer centers, Bowles et al showed that the risk of developing heart failure and/or cardiomyopathy (HF/CM) was higher in patients treated with trastuzumab compared to those treated with anthracyclines alone [2]. Cardinale et al. assessed the serum marker troponin-I pre- and post-dose in 251 patients receiving trastuzumab in the adjuvant and metastatic treatment of breast cancer [12]. This study revealed that troponin-I elevation occurred after initiation of trastuzumab in most patients, with subsequent trastuzumab-induced cardiotoxicity occurring one to eight months from the date of the first detectable troponin-I. This suggests that the intrinsic cardiotoxicity of trastuzumab is a problem and results in cardiomyocyte necrosis [12]. Troponin-I is considered a reliable marker of cardiac muscle tissue injury and is considered as a sensitive and specific biomarker in the diagnosis of myocardial infarction [13,14]. Unfortunately, there are no validated specific biomarkers for clinical use for early detection of trastuzumab-induced cardiotoxicity. Cardiac myosin light chain-1 (cMLC1) is a part of the myosin complex with an important role in cardiac muscle contraction. Impaired integrity of damaged or injured cardiomyocytes leads to release of cMLC1 from the myocardium into circulation [15-17]. It has not been reported if the release of cMLC1 from the myocardium into circulation is clinically related to trastuzumab-induced cardiotoxicity. 

Due to the lack of understanding of the molecular mechanisms of trastuzumab-induced cardiotoxicity, current clinical management relies only on the use of echocardiography to detect the reduction in LVEF [9,18]. Based on the extent of LVEF reduction, a decision regarding continuation or discontinuation of trastuzumab therapy is made [9]. However, reduction in LVEF is a part of the late phase of left ventricular dysfunction which occurs as a part of the heart compensatory mechanism to preserve contractility [18]. Biomarkers of trastuzumab-induced cardiotoxicity are needed for earlier detection and better management of critical early alterations. 

HER2 plays a critical role in cardiac development as has been shown in knock-out models [19,20]. Conditional ablation of HER2 in heart ventricle cells resulted in dilated cardiomyopathy in several studies [19,21] and increased sensitivity to anthracycline treatment [22]. Based on results from *in vitro* studies, trastuzumab-mediated suppression of HER2 signaling impairs the ability of cardiomyocytes to manage different types of stress, resulting in the loss in cardiomyocyte integrity [23,24]. Riccio, G et al have shown that trastuzumab binds to mouse HER2 and that mice treated with trastuzumab have reduced LVEF in their mouse models [25]. 

In this study, using echocardiography and electron microscopy, we aimed to evaluate the functional and ultrastructural consequences of trastuzumab treatment in mice, and by using microarray, the effects of trastuzumab on the expression of myocardial genes critically involved in cardiac and mitochondrial functions, adaptation to stress, and DNA repair. We also evaluated serum cMLC1 level as potential biomarker of trastuzumab-induced cardiotoxicity. 

## Results

### Trastuzumab altered the expression of genes involved in adaptability to cardiac contractility, hemodynamic stress, DNA repair mechanisms, apoptosis, and mitochondrial function

**Figure 1:**
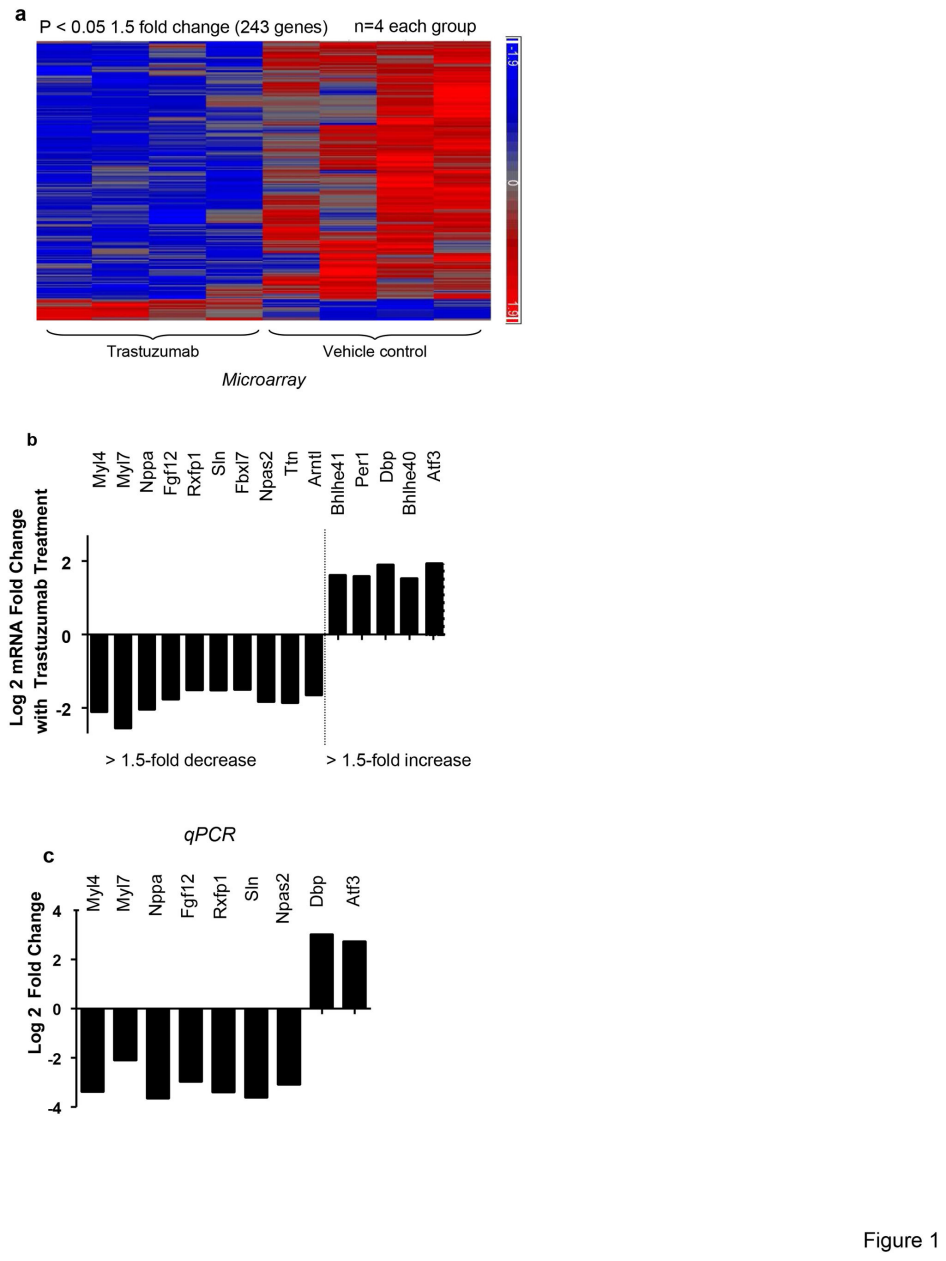
Trastuzumab altered the expression of genes that are essential for cardiac functions. (a) Heatmap representation of differentially expressed genes in the trastuzumab-treated animals compared to the control vehicle injected animals (blue = downregulation and red = upregulation); n=4 for each group. (b) Bar graph showing microarray results as Log_2_ of fold change. (c) Bar graph showing the qPCR validation of randomly selected genes from the microarray data. Results are shown as log_2_ of fold change. Analysis of variance was used to determine those probe sets significantly different between the two groups. The gene list was filtered with a fold-change cutoff of 2.

Previous studies have shown that trastuzumab can cause cardiac function decline in LVEF and fractional Shortening (FS) in mice [25,26]. Yet, the impact of trastuzumab on cardiomyocytes gene expression has not been reported. As shown in the representative heat map (Figure 1a), trastuzumab exerted an inhibitory effect on gene expression in mice treated with trastuzumab. At 1.5 fold change cutoff and an unadjusted P < 0.05, we found that 243 transcripts had a significantly different expression pattern in the trastuzumab-treated animal as compared to vehicle-treated animals (animals injected with the solvent only). Among these 243 genes, 229 were downregulated and 14 were upregulated (Figure 1a). Our analysis focused on the genes that changed by 1.5 fold or more and had an unadjusted P < 0.05. Pseudogenes, unclassified genes or genes with unknown function and short RNAs were excluded. Using that criterion, we identified 15 genes. Among these 15 genes, two distinct groups emerged. The first constituted nine genes *My14* [27],^*,*^ *My17* [28,29], *Nppa* [30], *Ttn* [31,32], *Rxfp* [33,34], *Sln* [35], *Fgf12* [36,37], *Fbx17* [38,39], *and Atf3* [40,41] have direct involvement in cardiac function, more specifically, cardiac contractility, adaptation to stress and hemodynamic pressure, as well as cardiomyocyte cellular function such as DNA repair, proliferation, wound healing and mitochondrial function. All of these genes except *Atf3* were significantly decreased in trastuzumab-treated animals (Figure 1b, Table 1). The second group included six genes (*Npas2* [42], *Dbp* [43,44], *Per1*[45] *Arntl* [42,46,47], *Bhlhe4* [48], *and Bhlhe40* [49]) that all modulate the circadian rhythm which influences heart rate, homeostasis, oxidative stress and mitochondrial function. *Npas2* and *Arntl* were downregulated; whereas *Dbp, Per1, Bhlhe41, and Bhlhe40* were upregulated (Table 1). Nine genes were randomly selected for Real-Time quantitative PCR confirmation. In all cases, the directionality of changes was confirmed and the magnitude of change was more than that observed in the microarray (Figure 1c). Table 2 showed the statistical analysis of qPCR data. 

**Table 1:** Microarray data showing the fold change in 15 genes.

Gene and protein name(s)	Fold change	P-value	Function and significance
Myl4, Myosin light polypeptide 4, Atrial myosin light chain 1 (ALC1)	-2.106	0.02800	Cytoskeletal motor protein; regulates atrial contractility and adaptation to hemodynamic pressure [27].
Myl7, Myosin light polypeptide 7, regulatory, Myosin light chain 2a (MLC2a)	-2.549	0.04900	Cytoskeletal motor protein; regulates atrial contractility and adaptation to stress [28,29].
Nppa, Atrial natriuretic peptide (ANP)	-2.038	0.01200	Adaptation to hemodynamic pressure; exerts anti-hypertensive and anti-hypertrophic properties on the heart through gene regulation [30].
Ttn, Titin, Connectin	-1.860	0.00050	Cytoskeletal protein; acts as a molecular spring that controls sarcomere length; functions in contractility and adaptation to hemodynamic stress; mutations in this gene are associated with dilated cardiomyopathy [31,32].
*Rxfp1*, Relaxin family peptide receptor 1	-1.510	0.02200	G-protein coupled receptor; functions in cardiovascular adaptive changes during pregnancy; the cognate ligand, relaxin, is an effective vasodilator and is considered a potential therapeutic agent for acute heart failure [33,34].
*Sln*, Sarcolipin	-1.520	0.02600	Sarcoplasmic reticulum transmembrane protein; regulates sacrcoplasmic reticulum calcium uptake and release in cardiomyocytes and its downregulation appears to result in aberrant Ca^2+^ uptake and atrial fibrillation [35].
*Fgf12*, fgf homologous factor 1 (FHF1), FGF 12	-1.760	0.02800	Cytoplasmic FGF homologous growth factor; attenuates cellular damage induced by ionizing radiation and is involved in cardiac sodium channels gating [36,37].
*Fbxl7*, F-box and leucine-rich repeat protein 7 (FBXL7)	-1.500	0.00005	E3 Ubiquitin ligase; regulates cell cycle; expression of *Fbxl7* variants correlate with increased risk of breast cancer in BRCA2 mutation carriers [38,39].
*Atf3*, Activating transcription factor 3 (ATF3)	+1.930	0.02200	Transcription factor; regulates gene expression that is induced by cardiac ischemia, associated with atrial enlargement, DNA damage, and degradation of mitochondria leading to apoptosis of cardiomyocytes [40]. In *Atf3* knockout mouse models, atrial enlargement and reduced LVEF occurs [41].
*Npas2*, Neuronal PER-acryl-hydrocarbon receptor nuclear translocator-SIM2 (Npas2), MOP4	-1.830	0.00050	Transcription factor; regulates circadian rhythm, regulate blood pressure, cardiovascular function, and vascular response to asynchronous stress [42].
*Dbp*, albumin D site-binding protein (DBP)	+1.900	0.00440	Transcription factor; regulates circadian rhythm, mediates apoptosis resulting from oxidative stress, and regulates blood pressure and cardiac hypertrophy [43,44].
*Per1*, Period homolog 1 (PER1)	+1.580	0.01170	Transcription factor; regulates expression of genes involved in circadian rhythm, sodium transport, and blood pressure [45].
*Arntl* (*Bmal1, MOP3*), Aryl hydrocarbon receptor nuclear translocator-like	-1.650	0.00170	Transcription factor; regulates circadian rhythm, blood pressure, heart rate, and mitochondrial metabolism [42,46,47].
*Bhlhe41* (*Dec2*), Basic helix-loop-helix family member e41	+1.610	0.00330	Transcription factor; regulates circadian rhythm and modulates hypertension susceptibility [48].
*Bhlhe40*, Basic helix-loop-helix family, member e40	+1.510	0.00036	Transcription factor; regulates circadian rhythm, and cardiac morphogenesis during development [49].

Microarray data showing the fold change in 15 genes with average expression level increased or decreased by more than 1.5-fold-change (P < 0.05). Four animals in each group were used (n=4).

**Table 2:** Statistical analysis of qPCR data.

***Gene***	***Fold Change Vs. Ctrl***	***P-Value***
Myl4	-3.372	0.054
Myl7	-2.083	0.165
Nppa	-3.639	0.017
Fgf12	-2.957	0.069
Rxfp1	-3.383	0.037
Npas2	-3.070	0.60
Sln	-3.596	0.053
Dbp	+3.012	0.004
Atf3	+0.368	0.072

Nine out of fifteen genes were selected for real-time quantitative PCR testing. In all case, the directionality of the changes was confirmed and the magnitude of changes was more than that seen in the microarray data. Nppa, Rxfp1, and Dbp showed statistically significance (P < 0.05).

### Trastuzumab treatment significantly reduced LVEF, FS, and the left ventricle posterior wall thickness

**Figure 2:**
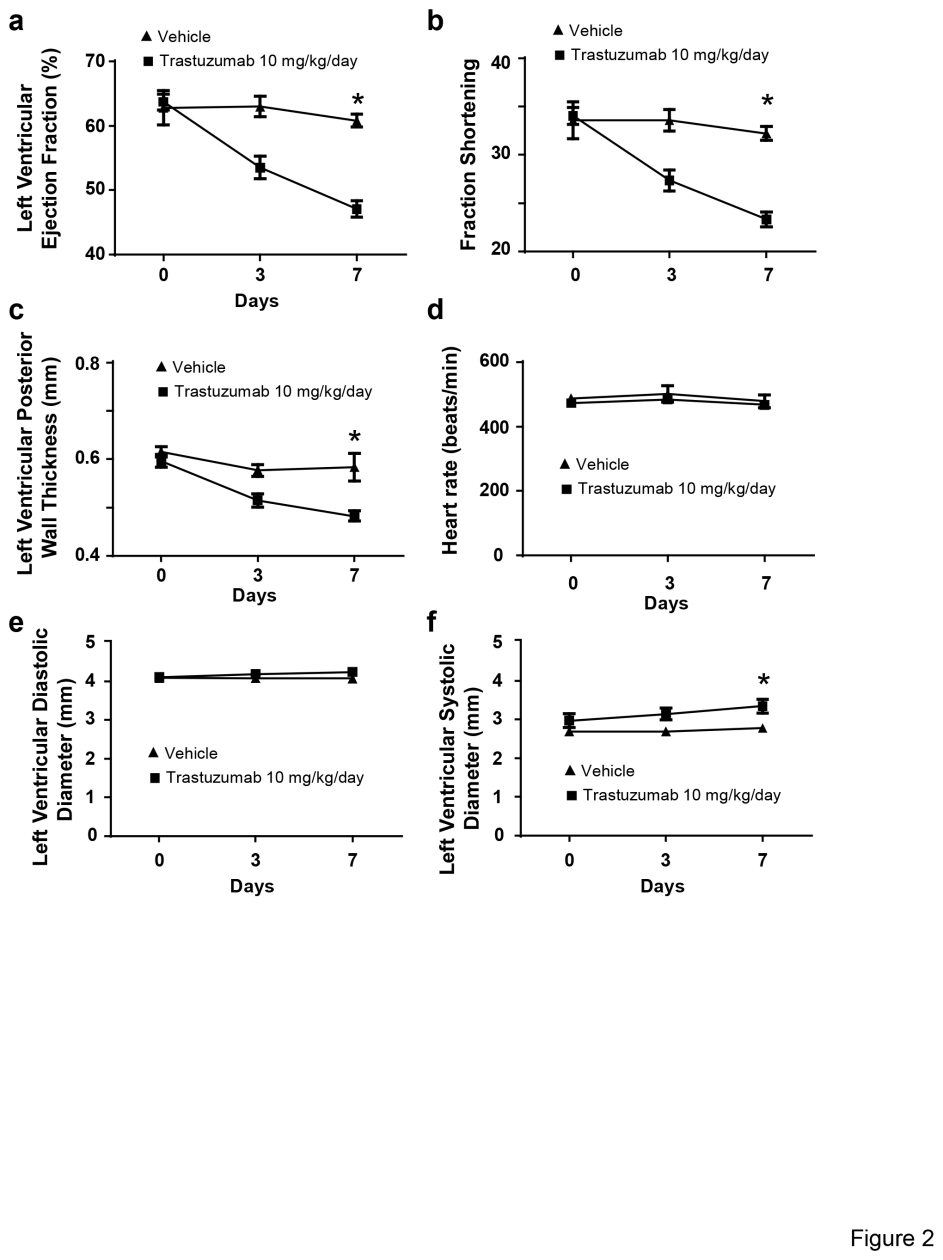
Trastuzumab impairs systolic function in mice. Mice received either trastuzumab (10mg/kg/day) (N = 10) or vehicle injections (N = 6). VEF (a),FS (b), heart rate (d), LVPW thickness (c), left ventricular systolic (f) and diastolic (e) diameter were evaluated at base line (day 0), and days 3 and 7 post injection. Data are presented as percentage of control animals (mean ± SEM). *P < 0.05 vs. the vehicle treated animals.

It has been reported that trastuzumab treatment can lead to a decline in LVEF and FS [9-11,25,26]. Our data also confirmed a decline in left ventricular function. By day seven after starting trastuzumab injection, LVEF declined from a mean of 61% in control animals to a mean of 47% in trastuzumab-treated animals (*P < 0.05) (Figure 2a). Fractional shortening (FS) also significantly declined from a mean of 32% in control animals to a mean of 23% in trastuzumab-treated animals (*P < 0.05). Control animals did not show significant fluctuation in their LVEF and FS (Figure 2a and 2b). There was a 17% decrease of the left ventricular posterior wall (LVPW) thickness in mice treated for 7 days with trastuzumab compared to vehicle, and also 19% increase in the left ventricular systolic dimension. In contrast, the heart rate and left ventricular diastolic dimensions were not altered by the treatment. The thickness of the posterior wall declined from a mean of 0.58 cm in control animals to 0.48 cm in trastuzumab-treated animals (*P < 0.05) (Figure 2c). The decline in LVEF and FS indicates that trastuzumab treatment potentially is associated with cardiac architectural damage impacting contractile capacity of the cardiomyocytes. Control animals did not show significant fluctuation in any of the hemodynamics parameters measured (Figure 2a-f).

### Trastuzumab treatment is associated with ultrastructural damages of heart tissues in mice

**Figure 3:**
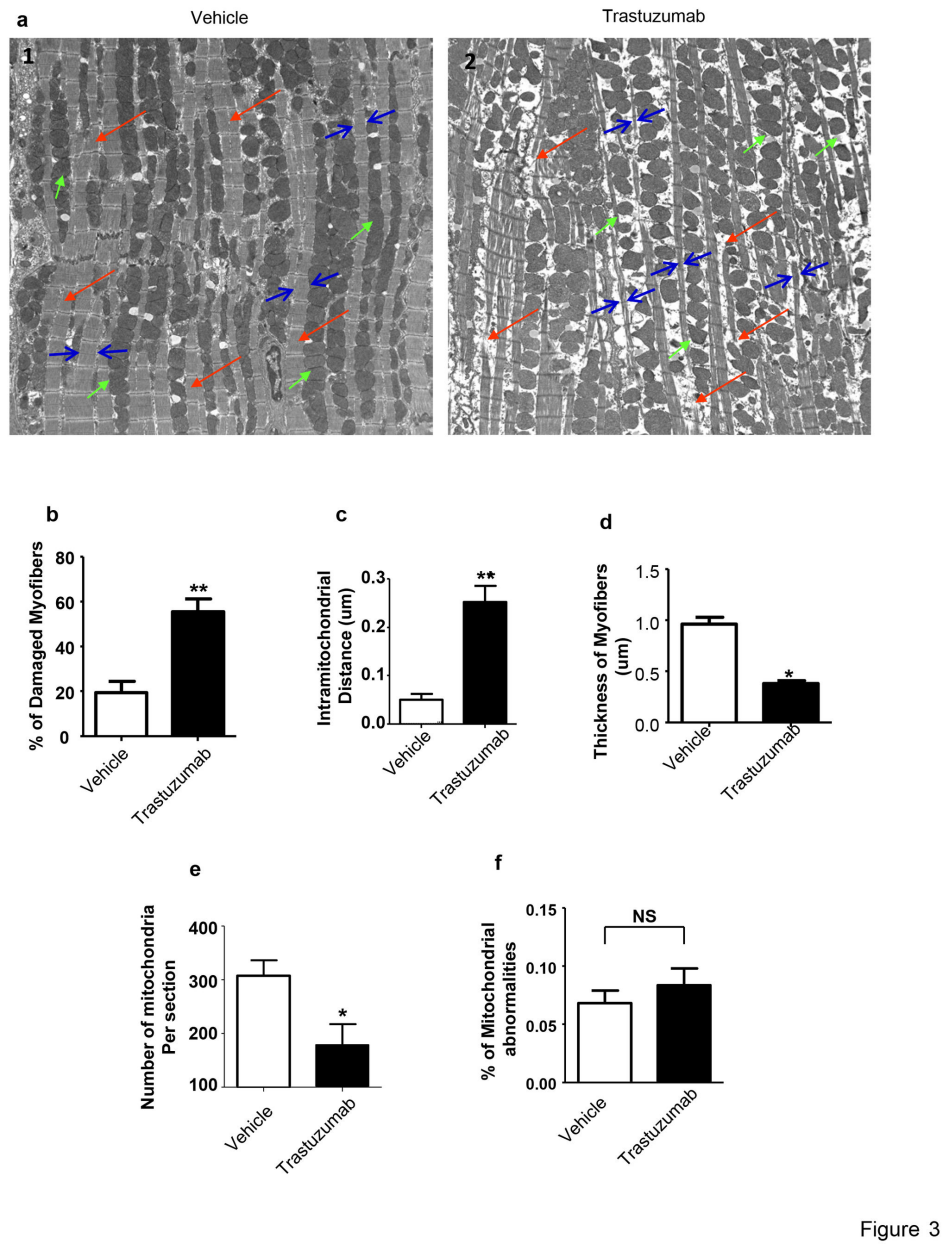
Electron microscopy imaging of cardiomyocyte ultrastuctures. Two mice from trastuzumab-treated and two mice from vehicle-treated groups were evaluated by EM. Animals were either treated daily with trastuzumab (10 mg/kg) or with vehicle control. On day 7, mice were euthanized and the hearts were harvested. See materials and methods for details of the fixation, embedding, and staining procedures. (**a**) A representative section of the left ventricle from a control mouse (**a1**) showing the typical mitochondrial density and intimate contacts (green arrows), connected myofibers (red arrows) and normal thickness of myofibers (pairs of blue arrows), compared to a section from trastuzumab-treated mice (**a2**) showing sporadic mitochondrial (green arrows), damaged disconnected myofibers (red arrows) and thinner myofibers (pairs of blue arrows). (**b**) Bar graph, quantification of damaged myofibers in trastuzumab-treated animals compared to control mice. Data are presented as a percentage of damaged and discontinued myofibers out of the total numbers of myofibers in the segment. (**c**) Bar graph showing a quantification of the distance between mitochondrial in trastuzumab-treated animals compared to control animals. Data presented in µm representing an average distance between mitochondria in the image. Magnification, 1000X (**d**) Bar graph showing a measurement of myofibers thickness in trastuzumab-treated animals compared to control mice. Data presented in µm representing the average thickness of myofibers. Magnification, 1000X. (**e**) Bar graph showing quantification of the number of mitochondria in sections from trastuzumab-treated animals compared to control mice. Data presented as average number of mitochondrial per segment. (f) Bar graph showing the percentage of damaged mitochondria (membrane disintegration, thinning of cristae and significant cavelae formation). In this figure, similar segments from the two animals in each group were used for this quantification. Student’s t-test was used to compare the two groups and significance is determined as *P < 0.05 or ** P < 0.01 vs. the vehicle treated animals.

We next tested whether trastuzumab treatment is associated with ultrastructural changes of the heart in mice treated with trastuzumab. As shown in Figure 3a, significant ultrastructural damages of heart tissues taken from the left ventricle were found in mice treated with trastuzumab (1). The cardiac myofibers (red arrows) were disconnected and damaged. The quantitative data for the percentage of damaged myofibers in mice treated with either trastuzumab or vehicle are showed in Figure 3b (2). Mitochondria in the cardiomyocytes of trastuzumab-treated animals lacked close contact that is the predominant organization of mitochondria observed in normal cardiomyocytes in the vehicle group (green arrows). Intermitochondrial distance (green arrows) was significantly increased in the cardiomyocytes from the left ventricles of trastuzumab-treated animals as compared to vehicle control animals (Figure 3c) (3). The cardiac myofibers appeared stretched with significantly reduced thickness in mice treated with trastuzumab as compared to vehicle control mice. This may reflect potential inhibition to their contractile function (blue arrows and Figure 3d) (4). The number of mitochondria per ventricular section (image frame) was significantly decreased in trastuzumab-treated animals compared to control animals (Figure 3e). Although a trend appeared to exist with mitochondrial abnormalities (mitochondrial membrane integrity compromised and/or mitochondrial cristae density changes), no significant differences were detected between the control and trastuzumab-treated animals (Figure 3f). The ultrastructural changes in the hearts of trastuzumab-treated mice might lead to diminished contractile potential of the heart. The ultrastructural damage to myofibers and changes in mitochondrial contact observed by EM highlighted the need to assess oxidative stress and cardiomyocyte viability. 

### Trastuzumab treatment induces oxidative stress in heart and results in increases in Caspase 3/7 activity

**Figure 4:**
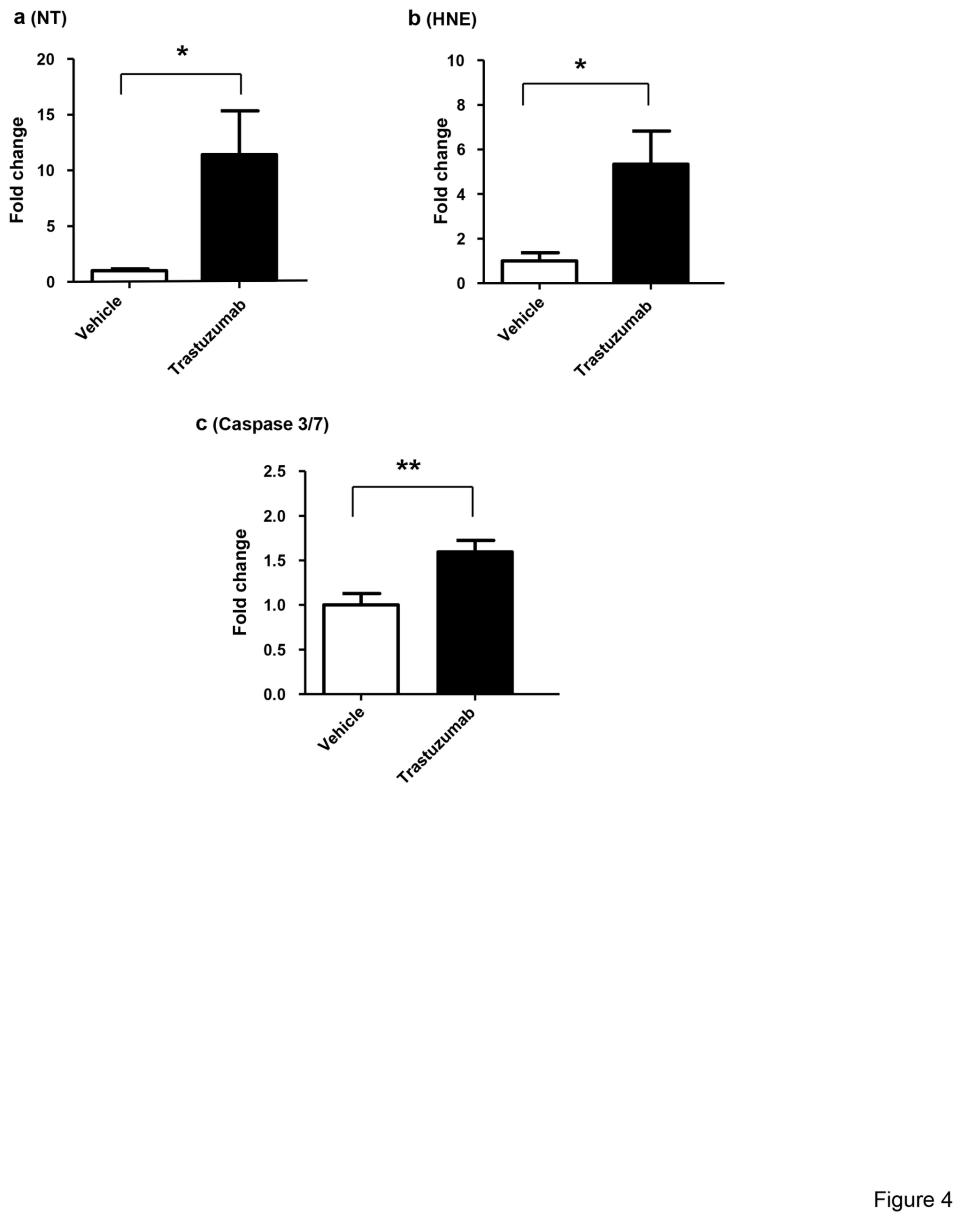
Trastuzumab induces oxidative stress and increases Caspase 3/7 activity in cardiomyocytes. (**a**) Quantification of ELISA measurement of NT in the hearts of animals treated with trastuzumab compared to the control vehicle injected animals. (**b**) Quantification of ELISA measurement of 4-HNE adducts in the hearts of animals treated with trastuzumab compared to the control vehicle injected animals. (**c**) Quantification of ELISA measurement of caspase 3 and 7 in the trastuzumab-treated animals compared to the control vehicle injected animals. For all the graphs in Figure 4, the results are expressed as fold changes relative to that of control vehicle-treated animals. Student’s t-test was used to compare the two groups and significance is determined as *P < 0.05 or **P< 0.01 vs. the vehicle treated animals. At least six animal were used for each group (n=6).

 It is established that treatment with anthracyclins, such as doxorubicin, lead to oxidative stress. As shown in Figure 4a, reactive nitrogen species formation, as indicated by 3-nitrotyrosine (NT) levels, was significantly elevated in the heart tissues of trastuzumab-treated animals (Figure 4a). 4-hydroxynonenal (4-HNE)-protein adducts were also significantly elevated by trastuzumab (Figure 4b). These results indicate that trastuzumab treatment results in oxidative stress. After confirming that trastuzumab treatment resulted in oxidative stress in heart, we assessed if the oxidative stress manifested in apoptosis in heart. As shown in Figure 4c, hearts from trastuzumab-treated animals showed a significant increase in Caspase 3/7 activity indicating the activation of apoptotic pathways. We additionally performed DNA fragmentation assays on heart tissues obtained from the mice treated with trastuzumab and control mice (data not shown). While up to a 20% increase in DNA fragmentation in mice treated with trastuzumab was observed as compared to that in the control mice, the differences in DNA fragmentation were not statistically significant. Taken together, these data suggested that trastuzumab-induced ultrastructural damages were likely caused by multiple mechanisms, including increased oxidative stress and possibly apoptosis. The ultrastructural damages induced by trastuzumab might lead to the release of components of cardiomyocytes contractile machinery into the blood stream. Such components might be potential biomarkers for trastuzumab-induced cardiac toxicity. We then evaluated serum levels of cardiac troponin-I and the cMLC-1 in mice treated with trastuzumab and control mice.

### Trastuzumab treatment increases serum levels of cardiac troponin-I (cTn-I) and cMLC1

**Figure 5:**
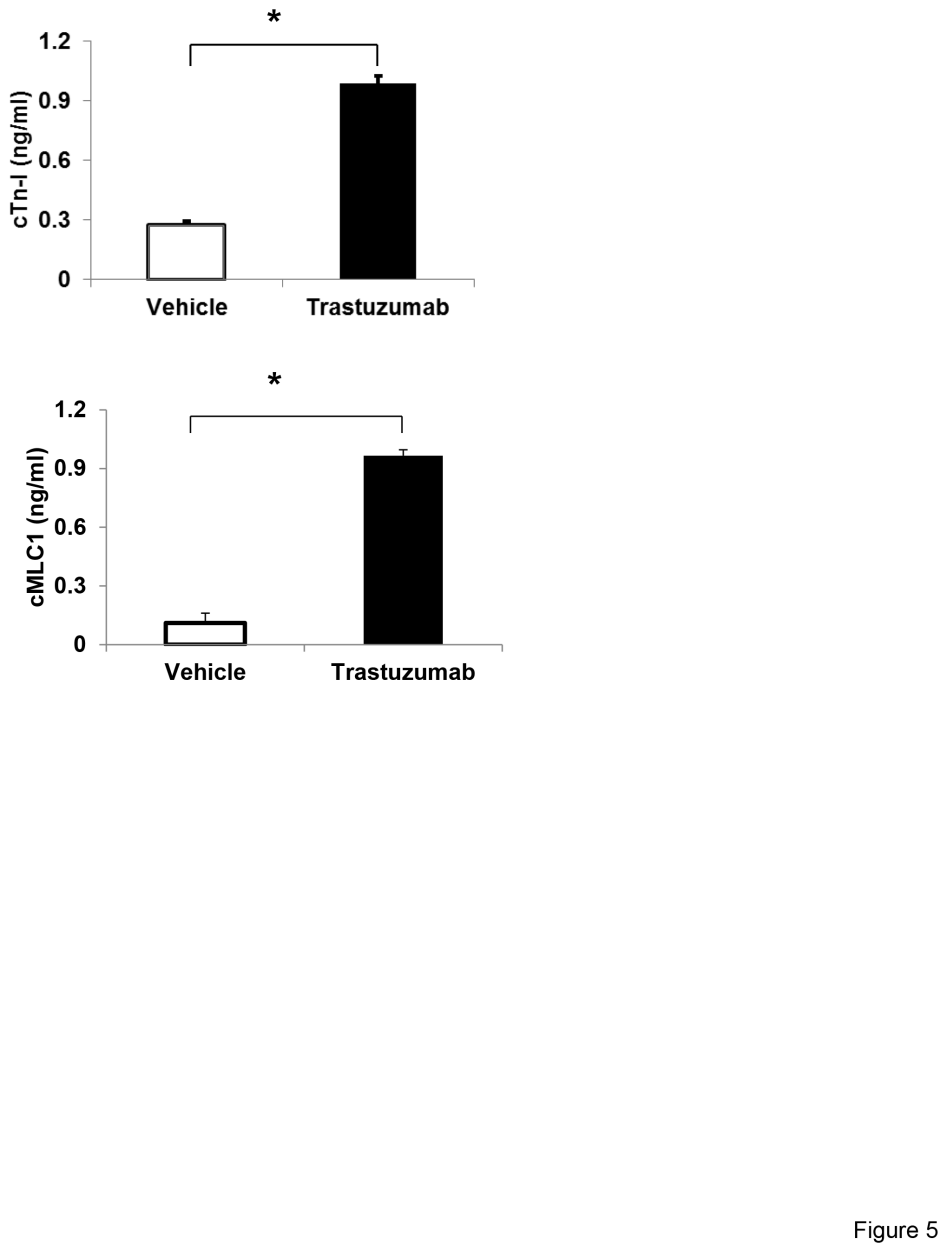
Effect of trastuzumab treatment on the levels of cTn-I and cMLC1 in mice sera. (**a**) The results of sandwich ELISA detecting the levels of cMLC1 in the serum of trastuzumab-treated mice compared to control mice injected with vehicle only. (**b**) The results of sandwich ELISA detecting the levels of cTn-I in the serum of trastuzumab-treated mice compared to control mice injected with vehicle only. Standard curve is constructed by plotting optical density (OD) values obtained from each reference point against its concentration in mg/mL. Absorbance values of vehicle and trastuzumab-treated mice samples are determined by corresponding concentrations from standard curve using linear regression analysis. Student’s t-test was used to compare the two groups. Significance is determined as *P < 0.05 vs. the vehicle treated animals. Six animal were used for each group.

It is well known that the blood levels of cardiac troponin-I (cTn-I) increase in response to cardiac toxicity. Troponin-I is a well-established biomarker of cardiomyocytes stress and damage. As shown in Figure 5a, cardiac troponin-I was significantly elevated in the serum of trastuzumab-treated mice as compared to that of control mice. We then assessed whether cardiac myosin light chain was released in the blood. As shown in Figure 5b, the serum levels of cMLC1 were significantly elevated in trastuzumab-treated mice as compared to that in control mice (Figure 5b). 

## Discussion

Treatment with trastuzumab is associated with considerable cardiotoxicity. However, the mechanisms of trastuzumab-induced cardiotoxicity remain elusive. In this study we provide evidence that trastuzumab treatment decreases the expression of myocardial genes that play a critical role in cardiac and mitochondrial functions, adaptation to stress, and DNA repair, which is associated with decline in left ventricular function and the thinning of the LVPW in mice, coupled with significant ultrastructural alterations in cardiomyocytes. We also show that trastuzumab treatment of mice increases myocardial oxidative and nitrative stress and activates apoptotic pathways, leading to elevations of serum troponin-I and cMLC1 levels. The elevated serum levels of cMLC1 in mice treated with trastuzumab highlights the potential that cMLC1 could be a useful biomarker for trastuzumab-induced cardiotoxicity. 

 We found that expression of *Myl4, Myl7* and *Nppa* decreased more than two-fold in trastuzumab-injected animals as shown by microarray. *My14* (MLC1a) is known to play an important role in ventricular adaptation to stressors such as pressure or volume overload ^27^. MLC1a also has been shown to increase troponin-I phosphorylation and to modulate cardiac contractility in response to hemodynamic demands [50]. *Myl7* (MLC2a) has been shown to be critical for atrial contractility and atrial function in general [28]. The importance of *Myl7* in the regulation of cardiac function is further highlighted by data demonstrating that *Myl7* inactivation is lethal in mice embryos due to cardiac complications [29]. *Nppa* (Natriuretic peptides-A precursor) is also demonstrated to play an essential role in adapting the heart to pressure overload by protective, antihypertensive, antihypertrophic effects on the heart [30]. Additionally, the thinning of the posterior wall might also reflect the lack of adaptability to the decline in LVEF and FS. *Ttn* (Connectin) is an essential part of the muscle sarcomeres as it connects microfilaments and is essential for the mechanical properties such as contractility and elasticity. Mutations in *Ttn* gene appear to be associated with dilated cardiomyopathy resulting from hemodynamic stress [31,32]. These findings are also consistent with our EM results demonstrating that significant ultrastructural damages of cardiomyocytes occur in mice treated with trastuzumab. The significance of *Rxfp1*, *Sln, Fgf12*, and *Fbxl7* genes in the regulation of cardiac, as well as cellular function can be found in Table 1. 

 Interestingly, the expression of *Atf3* was significantly upregulated. Turchi et al. reported that ATF3 protein contributes to UV-induced apoptosis through the regulation of hypoxia inducible factor (Hif)-2alpha expression, which in turn induces the expression of proapoptotic genes, such as Caspase7 or TRAIL [tumor necrosis factor (ligand) superfamily, member 10] [40] . They further reported that gain of function of Hif-2alpha and ATF3 protein was sufficient to trigger cell death, whereas loss of function of both proteins drastically inhibited UV-induced apoptosis [40] In addition, *Atf3* gene expression is associated with atrial enlargement and degradation of mitochondria leading to apoptosis of cardiomyocytes [41]. We demonstrated that trastuzumab treatment activated apoptotic pathways in cardiomyocytes. It will be interesting to investigate whether up-regulation of *Atf3* gene expression contributes to trastuzumab-induced apoptosis in cardiomyocytes. 

In summary, trastuzumab appears to interfere with genes important to cardiac function, adaptability to pressure, vasodilatation, and contractility (*Myl4, Myl7, Nppa, Rxfb1*and *Ttn*), genes that regulate calcium and sodium processing (*Sln* and *FGF12*), and genes that regulate DNA repair, mitochondrial function, and apoptosis (*Fbxl7* and *Atf3*). To the best of our knowledge, data presented in this study are the first to show the genetic alterations induced by trastuzumab in the myocardium using a mouse model.

 The expression of six transcription factors that modulate the circadian rhythm was also altered following trastuzumab injection. While we do not exactly know how these genes are involved in trastuzumab-induced cardiotoxicity, each has been shown to be involved in cardiac function. For instance, decreases in gene expression of *Npas2 or Arntl* may have an impact on blood pressure and vascular response to hemodynamic stress [42], and heart rate [46]. In addition, *Arntl* plays an important role in mitochondrial metabolism [47]. Although very few genes were shown to be significantly upregulated in the trastuzumab-injected animals by microarray (Figure 1a), four of the upregulated genes (*Bhlhe41, Per1, Dbp and Bhlhe40*) are known circadian rhythm modulators. *Bhlhe41* was shown to modulate hypertension susceptibility [45], *Per1* was found to play a role in regulating blood pressure [45], *Dbp* functions in cardiac hypertrophy, ventricular function, and contractility [43,51], while *Bhlhe40* functions in cardiac morphogenesis and development [49]. Moreover, *Dbp* appears to play a role in mediating apoptosis as a result of oxidative stress [44]. It is important to mention that these transcription factors regulate each other in well-known circadian clock feedback loops.

Trastuzumab enhanced myocardial levels of 4-hydroxynonenal (4-HNE) and 3-nitrotyrosine (NT) which are products of oxidative and nitrative stress. Trastuzumab treatment also resulted in increased activation of Caspases 3 and 7. Numerous experimental and clinical studies found increased production of reactive oxygen (superoxide, hydrogen peroxide, hydroxyl radical) and nitrogen (e.g. peroxynitrite) species in various forms of heart failure (TIPS). Increased nitroxidative stress can trigger mitochondrial dysfunction, inactivation of key contractile proteins, impairment of calcium homeostasis, activation of stress signaling, pathways involved in myocardial remodeling, such as matrix metalloproteinases, and cell death [52]. The complex interplay of nitroxidative stress with other secondary pathways (e.g. neuropeptides, neurohormones, cytokines, etc.) is involved in the deleterious way of the progression of cardiovascular dysfunction to heart failure, resulting in abnormalities in various cardiac receptors and signaling processes, calcium homeostasis, contractile proteins, besides structural alterations such as cardiovascular remodeling with hypertrophy, fibrosis, necrosis, and cardiac dilation [53]. Our results also suggest that nitroxidative stress may contribute to the cardiotoxicity of trastuzumab by promoting cell death and mitochondrial abnormalities/dysfunction, and by modulating the above mentioned pathological pathways. Taken together, our results indicate that trastuzumab treatment triggers cellular stress in cardiomyocytes resulting in the activation of pro-apoptotic machinery. 

Fallah-Rad et al recently reported that while 25 percent of HER2-positive breast cancer patients (10 out of 42 patients) developed trastuzumab-mediated cardiomyopathy during a three-year prospective clinical study, cardiac biomarkers such as troponin T, C-reactive protein, and brain natriuretic peptide did not change over time [54]. Sawaya et al also investigated whether early alterations of myocardial strain and blood biomarkers [Ultrasensitive troponin I, brain natriuretic peptide, and the interleukin family member (ST2)] predict incident cardiotoxicity in eighty-one women with newly diagnosed HER2-positive breast cancer treated with anthracyclines followed by taxanes and trastuzumab [55]. They found that systolic longitudinal myocardial strain and ultrasensitive troponin I measured at the completion of anthracyclines therapy might be useful in the prediction of subsequent cardiotoxicity and that no significant associations were observed for LVEF, brain natriuretic peptide, and ST2 [55]. cMLC1 and troponin-I are essential parts of the cardiac contractile machinery. Our data demonstrated that both proteins were elevated in the blood of trastuzumab-treated mice, indicating damage/loss of cardiomyocytes. In light of our results, it is worthwhile to investigate whether cMLC1 is detectable in the blood of trastuzumab-treated patients and to compare serum cMLC1 levels and time to detection to that of troponin-I. Such studies would determine whether cMLC1 could serve as a biomarker of trastuzumab-induced cardiotoxicity either alone, or in combination with troponin-I. 

Interestingly, a recent study by Barth et al did not find any alteration of left ventricular function in mice treated with trastuzumab (20mg/kg, biweekly intraperitoneal injections for three weeks) [56]. This may be due to different mouse strain (SCID-beige mice - the strain with immunodeficiency affecting both the B and T lymphocytes) and possibly a relatively lower dose of trastuzumab used in their study as compared to that used in ours, as well as others animal protocols [25]. 

 Collectively, we established a mouse model of trastuzumab-induced cardiotoxicity and demonstrated that the trastuzumab-induced cardiac dysfunction is associated with enhanced myocardial oxidative/nitrative stress and apoptosis, ultrastructural changes in cardiomyocytes, and altered myocardial expression of genes critically involved in cardiac and mitochondrial functions, adaptation to stress, and DNA repair. We also propose evaluation of serum cMLC1 serum level as a potential biomarker of trastuzumab-induced cardiotoxicity in humans. 

## Materials and Methods

### Animal model

The first batch of twenty C57/BL6 mice age 6-8 weeks (Jackson Laboratory, Bar Harbor, ME, USA) were randomly assigned to receive a daily intraperitoneal injection containing trastuzumab (Genentech, Inc), which was purchased from the pharmacy at the National Institutes of Health (NIH), Bethesda, MD, or the solvent provided by the manufacturer to dissolve the lyophilized trastuzumab (vehicle). Experiments were approved by and conducted in accordance with the regulations of the National Institute on Alcohol Abuse and Alcoholism (NIAAA) of NIH ACUC guidelines. Ten mice in the treatment group received 10 mg/kg/day of trastuzumab for six days and ten mice in the control group received a comparable volume of vehicle. Mice were subjected to echocardiography procedure (ECHO) on day one prior to the first injection establishing a base-line for cardiac performance and then on day three and finally on day seven directly prior to blood collection and euthanasia to harvest the hearts. The second batch of sixteen C57/BL6 mice was divided into two groups. The trastuzumab treatment group and vehicle control group were comprised of eight mice each and treated exactly as described above, but no echocardiography was performed. Hearts were isolated on day seven for both groups and used for the oxidative stress and apoptosis assays described below. 

### Genome-wide mRNA expression microarray analysis

Total RNA from whole hearts collected from four animals per group (vehicle or trastuzumab) in the first batch of mice was isolated and purified using QIAGEN RNeasy fibrous tissue kit (QIAGEN, Valencia, CA Cat#74704). RNA quality was ensured using the Agilent RNA 6000 Nano kit and the Bioanalyzer 2100. 150 ng of total RNA was used to generate sense-strand cDNA by The Ambion WT Expression Kit (Applied Biosystems). The fragmentation and terminal labeling were done using the Affymetrix GeneChip WT Terminal Labeling Kit (Affymetrix, Santa Clara, CA). Approximately 25ng/ul concentration of cDNA was hybridized to each Affymetrix Mouse Gene 1.0 ST Array Chip. The arrays were washed and stained using the fluidics protocol FS450_0007 procedure on an Affymetrix Fluidics Station 450. The probe intensities were scanned by the GeneChip Scanner 3000. The raw data were normalized and analyzed using Partek Genomic Suite^®^ (Partek Inc., St Louis, MO, USA). Analysis of variance was used to determine those probe sets significantly different between the two groups. The gene list was filtered with a fold-change cutoff of 1.5. 

### Real-time quantitative PCR

 cDNA used in the real-time quantitative PCR was made from the same RNA isolated for the microarray. Nine out of fifteen identified genes were randomly selected for quantitative PCR confirmation. Quantitative PCR was performed using Taqman Fast Universal PCR Master Mix and commercially available primers for genes with the 7900 Fast Real-Time PCR system (Applied Biosystems, Foster City, CA). GAPDH (glyceraldehyde-3-phosphate dehydrogenase) gene was used as reference to normalize the expression level. After normalization, GUSB (glucuronidase, beta) had similar expression levels among all of the samples. 

### Echocardiography (ECHO)

Echocardiography was performed using a Vevo 770 ultrasound system (Visualsonics, Toronto, Canada) equipped with a real time micro-visualization scan head probe working at a frame rate ranging between 110 and 120 frames per sec (fps). The nosepiece-transducer used had a central frequency of 30 MHz, a focal length of 12.7 mm and 55 µm of nominal spatial resolution. The Vevo 770 is equipped with ECG-gated kilohertz visualization software (EKV™), which synthesizes high temporal resolution B-Mode images by combining several ECG-synchronized heart cycles. The echocardiogram reconstruction software produces B-mode sequences at up to 1000 frames per second. Mice were anesthetized by 2% isoflurane mixed with 0.5 L/min 100% O_2_ and placed on a warming pad (37°C). Moisturizer gel was applied to extremities prior to using tape to fix the mouse to the base plate. The plate was heated and allowed for a tight control of body temperature. Continuous application of anesthesia was insured through the flow of 1.0% to 1.5% isoflurane mixed with 0.5 L/min 100% O_2_ into a rubberized tube fixed to the snout. The left ventricular FS, LVEF, LVPW, heart rate, left ventricular diastolic diameter, left ventricular systolic diameter were measured and assessed. .

### Electron microscopy

Left ventricle specimens were fixed for 1 hour at room temperature and then overnight at 4 °C in 4% formaldehyde, 2% glutaraldehyde, 0.1 M cacodylate buffer (pH 7.4), osmicated for 1 hour at room temperature in the same buffer, en bloc stained with 0.5% uranyl acetate for 1 hour, dehydrated in a graded ethanol series, embedded in Epon 812 substitute, and examined on a Hitachi H7650 transmission electron microscope. The number of mitochondria was calculated from segments from each animal. Segments analyzed were at the same magnification and from similar anatomical regions. Intermitochondrial distance and myofibers thickness were measured using ImageJ® software.

### Apoptosis and oxidative stress

Harvesting of heart tissue: The chest cavity of anesthetized mice from the second batch of the mice was opened and tissue surrounding the heart was cleared, including vasculature and pericardium. Hearts were flushed using 2.0 ml of PBS then placed in a microtube and snap frozen in liquid nitrogen. 

Caspase 3/7 ELISA: **Promega**’s Apo-ONE^®^ Homogeneous Caspase-3/7 assay ( G7791) was used to detect caspase activity in the hearts of mice from each group (vehicle or trastuzumab) in the second batch of the mice. The experiments were performed according to manufacturer’s instructions. 

Hydroxynonenal detection ELISA: OxiSelect^TM^ HNE Adduct ELISA kit (Cell Biolabs, INC. Cat# STA-338) was used to detect oxidative cellular damage in the hearts of mice from all groups. The assay was performed according to the manufacturer’s instructions. 

Nitrotyrosine detection ELISA: OxiSelect^TM^ Nitrotyrosine ELISA kit (Cell Biolabs, INC. Cat# STA-308) was used for the detection of modification of tyrosine residues in proteins to 3-nitrotyrosine by peroxynitrite or other nitrating agents as a product of oxidative stress. The assay was performed according to the manufacturer’s instructions.

### Assays for serum biomarkers

Blood collection and serum isolation: To avoid any puncture to the major vessel or heart, retro-orbital blood collection was performed on six mice from each group (vehicle or trastuzumab) in the first batch of the mice. Blood was collected at the time of euthanasia in T-MG microtubes (Terumo Medical Corporation^®^) with a clot-activating gel barrier allowing for serum collection after short centrifugation. 

Cardiac troponin-1 (cTn1) ELISA: Cardiac troponin-I levels were measured by sandwich ELISA using the high sensitivity cTnl ELISA (Life Diagnostics, Inc Cat# 2010-1-HS) that recognizes a specific epitope on mouse cardiac Troponin-I (cTn1). Each serum sample was assayed in triplicate.

Cardiac myosin light chain-1 (cMLC1) ELISA: **S**andwich ELISA was utilized to detect the cardiac isoform of MLC1 levels in mouse sera (Life Diagnostics, Inc Cat# 2320-2). The assays were performed according to the manufacturer’s protocol. Each serum sample was assayed in triplicate. 

### Statistical analysis

GraphPad Prism 5^©^ was used to perform statistical analyses and to plot the data. Data were analyzed by Student's t-test with a P < 0.05 considered significant. P < 0.05 was marked as (*) and a P < 0.01 marked as (**). Data were expressed as mean ± SEM.
