# Correction: Trastuzumab Alters the Expression of Genes Essential for Cardiac Function and Induces Ultrastructural Changes of Cardiomyocytes in Mice

**DOI:** 10.1371/annotation/3ba18ef8-8c9c-45ab-9bc5-ad571a54a28c

**Published:** 2014-01-06

**Authors:** M. Khair ElZarrad, Partha Mukhopadhyay, Nishant Mohan, Enkui Hao, Milos Dokmanovic, Dianne S. Hirsch, Yi Shen, Pal Pacher, Wen Jin Wu

In the Funding Statement, the name of a funding organization is incorrect. The Funding Statement should read: "This study is supported by Food and Drug Administration-Office of Women’s Health Research Science Program Award to WJW (Project ID: 750912CDR) and the Interagency Oncology Task Force Joint Fellowship Program sponsored by the United States Food and Drug Administration and National Cancer Institute. The funders had no role in study design, data collection and analysis, decision to publish, or preparation of the manuscript." 

